# Online-risks and user activity in Russian adolescents: Comparing results from 2010-2019 Russian population studies

**DOI:** 10.1192/j.eurpsy.2021.555

**Published:** 2021-08-13

**Authors:** G. Soldatova, E. Rasskazova, A. Koshevaya

**Affiliations:** Faculty Of Psychology, Lomonosov Moscow State University, Moscow, Russian Federation

**Keywords:** online-risks, user activity, adolescents, population studies

## Abstract

**Introduction:**

Internet provides wide opportunities not only for development, but also for developmental risks including information about drug consumption, advocacy for weight loss and suicide (Livingstone et al., 2011).

**Objectives:**

The aim of this study was to compare user activity and online risks in Russian adolescents and parents according to three population studies, 2010, 2013, and 2019.

**Methods:**

Using EU Kids Online methodology (Livingstone et al., 2011), the data of 1219 parents of adolescents 12-17 years old and 1553 adolescents 12-17 years old from 15 regions of Russia in 2019 were compared with the data of 1203 adolescents 12-17 years old and 1209 parents in 2013, as well as the answers of 685 couples “parent - teenager 12-16 years old” in 2010.

**Results:**

Compared to 2010, in 2019 adolescents are more frequently disappointed or worried about something online (41.5% versus 33.6%, χ^2^=12.44, p<.01, CC=.07). Contemporary adolescents rarer report having seen sexual images (χ^2^=339,48, p<0,01, CC=0,36) online but more frequently report having seen rude actions online (χ^2^=69,93, p<0,01, CC=0,16). They more frequently see information about drugs and suicides (χ^2^=37,11, p<0,01, CC=0,12 и χ^2^=85,04, p<0,01, CC=0,17, respectively) and report an experience of cyberaggression (χ^2^=80,85, p<0,01, CC=0,19) and fraud (χ^2^=17,75, p<0,01, CC=0,09).

**Conclusions:**

In Russian sample online risks in adolescents closely related to mental health and risky behavior in 2010-2019 increase suggesting necessity of social programs of risk prevention. Study is supported by Russian science Foundation, project 18-18-00365.

**Conflict of interest:**

Study is supported by Russian science Foundation, project 18-18-00365.

